# Calcium Hides the Clue: Unraveling the Diagnostic Value of Coronary Calcium Scoring in Cardiac Arrest Survivors

**DOI:** 10.3390/jpm15090422

**Published:** 2025-09-03

**Authors:** Ana Margarida Martins, Joana Rigueira, Beatriz Valente Silva, Beatriz Nogueira Garcia, Pedro Alves da Silva, Ana Abrantes, Rui Plácido, Doroteia Silva, Fausto J. Pinto, Ana G. Almeida

**Affiliations:** 1 Serviço de Cardiologia, Departamento de Coração e Vasos, ULS Santa Maria, Hospital de Santa Maria, Av Prof. Egas Moniz, 1649-028 Lisboa, Portugalana.n.garcia@ulssm.min-saude.pt (B.N.G.); pmsilva@edu.ulisboa.pt (P.A.d.S.); ana.l.abrantes@ulssm.min-saude.pt (A.A.); fausto.pinnto@ulssml.min-saude.pt (F.J.P.);; 2 Serviço de Medicina Intensiva, ULS Santa Maria, Hospital de Santa Maria, Av Prof. Egas Moniz, 1649-028 Lisboa, Portugal

**Keywords:** coronary artery calcium, out of hospital cardiac arrest

## Abstract

**Introduction**: Coronary artery disease remains one of the most prevalent causes of hospital cardiac arrest (OHCA). Although the benefit of early coronary angiography is well stablished in patients with ST-segment elevation, the benefit and the timing of performing it in other patients remain a matter of debate. This is due to the difficulty of identifying those in which an infarction with non-ST-segment elevation is the cause of the OHCA. Coronary artery calcium (CAC) emerges as a reliable predictor of coronary disease and adverse cardiovascular events, detectable even in non-gated chest computed tomography (CT) scans commonly used in OHCA etiological studies, showcasing potential for streamlined risk assessment and management. **Aim**: The aim of this study was to evaluate if CAC in non-gated CT scans performed in OHCA survivors could act as a good predictor of coronary artery disease on coronary angiography. **Methods**: This is a single-center, retrospective study of OHCA survivors without ST-segment elevation at presentation. We selected patients for whom a non-gated chest CT was performed and underwent coronary angiography due to the clinical, electrocardiogram (ECG), or echocardiographic suspicion of acute coronary syndrome. An investigator, blinded to the coronary angiography report, evaluated CAC both quantitively (with Agatston score) and qualitatively (visual assessment: absent, mild, moderate, or severe). **Results**: A total of 44 consecutive patients were included: 70% male, mean age of 60 ± 13 years old. The mean Agatston score was 396 ± 573 AU (Agatston units). Regarding the qualitative assessment, CAC was classified as mild, moderate, and severe in 11%, 25%, and 20% of patients, respectively. The coronary angiography revealed significant coronary lesions in 15 patients (34%), of which 87% were revascularized (80% underwent PCI and 7% CABG). The quantitative CAC assessment accurately predicted the presence of significant lesions on coronary angiography (AUC = 0.90, 95% CI 0.81–0.99, *p* < 0.001). The presence of moderate or severe CAC by visual assessment also predicted significant lesions on coronary angiography (OR 2.66, 95% CI 1.87–109.71, *p* = 0.01). There was also a good and significant correlation between the vessel with severe calcification in the CT scan and the culprit vessel evaluated by coronary angiography. CAC was reported in only 16% of the reviewed CTs, most of them with severe calcification. **Conclusion**: The assessment of CAC in non-gated chest CT scans proved to be feasible and displayed a robust correlation with the presence, severity, and location of coronary artery disease. Its routine use upfront was shown to be an important complement to CT scan reports, ensuring more precise and personalized OHCA management.

## 1. Introduction

The prognosis of out-of-hospital cardiac arrest (OHCA) remains dismal, even for patients successfully resuscitated and admitted to hospital, with mortality rates as high as 74% [1]. Obstructive coronary artery disease is a predominant factor in most adult cardiac arrest (CA) cases, making acute coronary syndrome an essential consideration in differential diagnosis [2,3]. Although dedicated clinical trials are lacking in this specific context, patients with the return of spontaneous circulation (ROSC) and persistent ST-segment elevation should, in general, undergo immediate coronary angiography based on the overall clinical situation and a reasonable benefit/risk ratio [4,5,6].

Within the larger subset of patients without ST-segment elevation, the etiological spectrum is notably diverse, encompassing both cardiac and noncardiac factors. In this subset of patients, the benefits of early coronary angiography are increasingly debated [7]. On the one hand, in patients with myocardial infarction, early revascularization can reduce infarct size, preserve ventricular function, and lower the risk of adverse outcomes associated with myocardial injury, such as heart failure or arrhythmias. On the other hand, routine early coronary angiography carries potential drawbacks, including procedural risks, delays in identifying and treating causes of cardiac arrest unrelated to acute coronary syndrome, and the possibility of postponing neuroprotective interventions.

Coronary artery calcium (CAC) is a reliable predictor of coronary artery disease and adverse cardiovascular events [8,9]. Although the conventional method for assessing coronary artery calcium involves an electrocardiography (ECG)-gated computed tomography (CT) scan with standard reconstruction and acquisition parameters, its detection is also feasible during non-gated chest CT examination [10], such as those routinely established for etiological study in OHCA survivors. Recent studies highlight a strong association between the CAC scores obtained from non-gated chest CT scans and formal CAC testing, as well as coronary angiography [11].

The aim of this study was to evaluate if CAC documented in non-gated CT scans performed in OHCA survivors could act as a good predictor of obstructive coronary artery disease on coronary angiography. That would help to select those without ST elevation at baseline, who could benefit from an early revascularization approach, thus preventing unnecessary procedures or critical delays, enabling better personalized care in this population with very high mortality risk.

## 2. Methods

This was a single-center retrospective study performed at a tertiary hospital (Unidade Local de Saúde de Santa Maria, Lisbon, Portugal) from 1 January 2017 to 31 December 2022. We selected patients aged ≥18 years who were admitted after OHCA and successful resuscitation and that either underwent a non-gated chest CT as a part of etiological study at admission or had a previous chest CT (up until 6 months before the CA event). In addition, patients were included only if they underwent coronary angiography due to the suspicion of acute coronary syndrome during the current hospitalization in order to understand the coronary anatomy and thus enable a comparison with the data obtained from the chest CT. Patients with a previous history of percutaneous coronary intervention (PCI) or coronary artery bypass graft (CABG) were excluded from the analysis, since CAC has no validation in this population and may be overestimated. Patients who presented with ST-segment elevation were also excluded since OHCA etiology was immediately assumed, obviating the need for a further differential diagnosis at presentation. Patient data regarding baseline demographics, clinical presentation, medical history, laboratory values, electrocardiographic, echocardiographic, and procedural data were extracted from the electronic medical records. All data were retrospectively collected by the authors. Due to the retrospective analysis of the data, the need for informed consent was waived by the institution.

### 2.1. Chest Computed Tomography Protocol and Coronary Artery Calcification Evaluation

The CT scans were taken using a 64-slice or 16-slice multidetector CT system (Siemens Medical Solutions USA Inc, Malvern, PA, USA). Imaging acquisition included axial slices with a slide thickness of 3 mm, with parameters adjusted to optimize spatial resolution and reduce motion artifacts. CT studies were analyzed without additional processing using Patient Archiving and Communication System (PACS) software (Phillips IntelliSpace Portal 8.0), and post-acquisition processing was performed in axial planes.

The assessment of CAC was conducted by an investigator blinded to the coronary angiography report. To identify and quantify CAC, we used a quantitative method employing the Agatston score with a conventional 130 HU threshold. This score was calculated independently for each of the main coronary artery branches (left main artery [LM], left anterior descending artery [LAD], circumflex artery [CX], and right coronary artery [RCA]), and the obtained sum was used to create a score reflecting the global coronary calcium burden. For risk stratification, the Agatston score was further classified into three risk categories based on previous studies: no CAC (0), mild CAC (1–100), moderate CAC (100–400), and severe CAC (>400). We also used a qualitative assessment involving a visual quantification of the extent of global coronary artery tree calcification, categorized as absent (0 points), mild (1 point), moderate (2 points), or severe (3 points).

We reviewed the CT report to ascertain the presence of any mention of CAC in the text’s body and/or conclusion. A report was deemed to include CAC if any reference to coronary calcification or atherosclerosis was identified. References to valvular or aortic calcification, indeterminate vascular calcifications, or non-coronary atherosclerotic disease were excluded as criteria for the existence of CAC reporting.

### 2.2. Coronary Angiography Evaluation

Coronarography performed in the same admission was evaluated, and the reports were analyzed. The left and right coronary arteries were accessed using standard fluoroscopic views (cranial and caudal views, left and right). Coronary lesions > 70% in any of the three main epicardial vessels or >50% in the left main artery were deemed significant.

### 2.3. Statistical Analysis

Categorical variables were expressed as frequency counts and percentages, and continuous variables were expressed as the mean and standard deviation (SD). The Shapiro–Wilk test or skewness and kurtosis were used to test the distributions for normality. Frequency tables were obtained, and data analysis was performed with an independent samples *t*-test or the Mann–Whitney U-test to compare continuous variables and with the chi-square test or Fisher’s exact test to compare categorical variables, as appropriate based on the distribution. Cohen’s kappa coefficient was used to measure correspondence between the vessel with at least severe calcification in the CT scan and the vessel identified as the culprit in the coronary angiography. A receiver-operating characteristic (ROC) curve was plotted, and the area under the ROC curve (AUC) was calculated as an index for the accuracy of the prediction (discriminatory power) of the model.

Statistical significance was defined as a *p* value < 0.05. The statistical software used to analyze the data was SPSS^®^ v.26 (IBM).

## 3. Results

**Figure 1:**
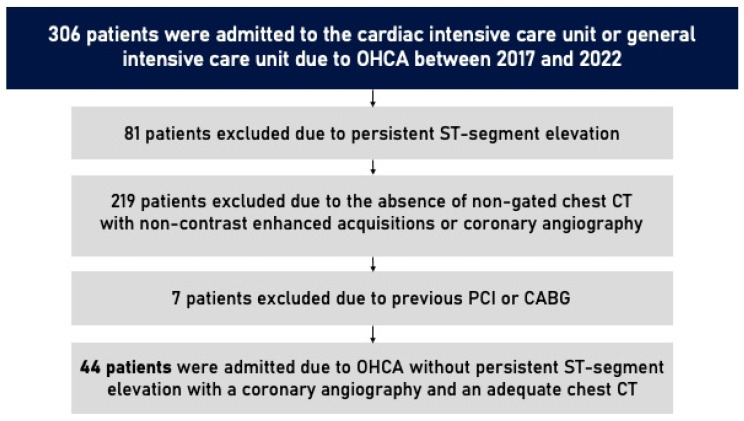
Study flowchart. OHCA, out-of-hospital cardiac arrest; CT, computed tomography; PCI, percutaneous coronary intervention; CABG, coronary artery bypass graft.

A total of 44 patients were included in this study. From 1 January 2017 to 31 December 2022, 306 patients were admitted to the cardiac intensive care unit or general intensive care unit in CHULN due to OHCA. In total, 81 patients were excluded due to persistent ST-segment elevation. Of the 225 who remained, only 51 underwent a non-gated chest CT with non-enhanced acquisitions as a part of an etiological study of OHCA or had a previous non-gated non-contrasted chest CT (up until 6 months before the event) and underwent coronary angiography due to the clinical suspicion of acute coronary syndrome. Seven patients were excluded due to previous PCI or CABG. The study flowchart is shown in Figure 1.

Most chest CTs were performed as a part of the etiological investigation of CA (n = 41, 93%). In three cases, we used previous CTs performed due to suspected respiratory infection and lung cancer screening.

The baseline characteristics of the cohort are described in Table 1. The mean age was 60 years (±13), with 70% of patients being male. The most prevalent comorbidity was arterial hypertension (52%), followed by dyslipidemia (32%). More frequently, CA occurred at home (43%), and in 95% of cases it was witnessed, with a mean no-flow time of 4.1 min and a low-flow time of 18.4 min. Most patients presented with a shockable rhythm (59%).

CT scans were performed at a mean interval of 3.7 h after first medical contact. Coronary artery calcium was identified in 25 (57%) patients. The mean Agatston score was 396 ± 573 AU. In total, 4 patients (9%) showed mild CAC (Agatston score 1–100), 5 patients (11%) showed moderate CAC (Agatston score 100–400), 16 patients (36%) exhibited severe CAC (Agatston score > 400), and the remaining 19 had no CAC. Regarding the qualitative assessment by the visual quantification of the extent of global coronary artery calcification, 19 patients (43%) were categorized as absent (0 points), 5 patients (11%) as mild (1 point), 11 patients (25%) as moderate (2 points), and 9 patients (21%) presented severe (3 points) calcification.

The coronary angiography revealed significant coronary lesions in 15 patients (34%) –3 (7%) in LM, 13 (30%) in LAD, 7 (16%) in Cx, and 3 (7%) in RCA. Of those, 13 patients (87%) were revascularized (80% underwent PCI and 7% CABG). Patients with significant lesions on coronary angiography exhibited a higher prevalence of cardiovascular risk factors, including arterial hypertension (67% vs. 45%, *p* = 0.213), dyslipidemia—defined as the previous use of anti-lipidemic therapies or elevated cholesterol levels in relation to CV risk estimation—(47% vs. 24%, *p* = 0.177), diabetes mellitus (40% vs. 7%, *p* = 0.013), and smoking (33% vs. 24%, *p* = 0.722), although only diabetes reached statistical significance. No significant differences were observed regarding the characteristics of CA (Table 1).

Patients without significant lesions on coronary angiography exhibited significantly lower Agatston scores compared to those with significant lesions (136 [±270] vs. 899 [±671], *p* < 0.001). Similarly, regarding the visual assessment of coronary artery calcium (CAC), patients without lesions on coronary angiography had a median CAC visual assessment score significantly lower than those with significant lesions (0 [IQR 0–1] vs. 2 [IQR 2–3], *p* < 0.001). Considering the patients who showed an Agatston score of 0 (n = 19, 43.2%), none showed significant lesions on coronary angiography. On the other hand, 68.7% (n = 11) of patients with Agatston score >400 had significant lesions on coronary angiography.

**Figure 2:**
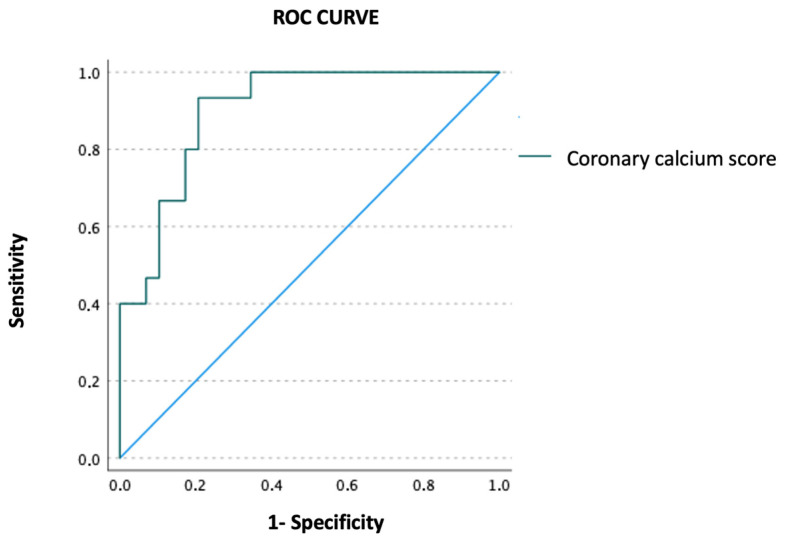
Receiver-operating characteristic (ROC) curve illustrating the diagnostic performance of the quantitative coronary artery calcium score (Agatston score) in predicting the likelihood of significant lesions detected during coronary angioplasty.

Using ROC curve analysis, quantitative CAC assessment accurately predicted the presence of significant lesions on coronary angiography (AUC = 0.901, 95% CI 0.814–0.988, *p* < 0.001) (Figure 2). Moreover, the presence of moderate or severe CAC by visual assessment also predicted significant lesions on coronary angiography (OR 2.66, 95% CI 1.87–109.71, *p* = 0.010).

As demonstrated in Table 2, patients with significant lesions on coronary angiography exhibited notably higher coronary artery calcium (CAC) scores for the corresponding vessel, with the exception of the left main artery. There was also a substantial and significant correlation between the vessel with severe calcification in CT scan (Agatston score > 400) and the degree of vessel stenosis evaluated by coronary angiography (kappa = 0.615, *p* < 0.001).

CAC was reported in only 16% of the reviewed CTs, most of them with severe calcification. In patients with severe calcification (by visual assessment), only 4 out of 9 reports mentioned it. One report specified the location of CAC, but none mentioned the absence of CAC. Among the patients who underwent coronary angiography and were revascularized, coronary calcification was present in the CT scan report of only 50% of them.

## 4. Discussion

This study emphasizes the potential usefulness of non-gated CT scans in identifying OHCA survivors at higher risk of significant coronary artery disease, thereby pinpointing those who would benefit most from an early coronary angiography strategy, eliminating potentially critical delays and ensuring a better personalized care in such an high mortality setting.

Despite advancements in resuscitation and intensive care management, the prognosis for OHCA remains unfavorable [1]. Following successful resuscitation, optimal care entails targeted temperature management, vital organ support, and addressing the underlying cause of the arrest. Determining the cause of OHCA is often challenging due to the unavailability of medical history and the complexity of interpreting complementary exam results, leading to ambiguity regarding the appropriate course of treatment.

The opportunity to address the underlying cause of CA through coronary revascularization holds significant promise in preventing recurrence, promoting cardiac recovery, managing or preventing cardiogenic shock, and mitigating long-term complications such as heart failure. Numerous observational studies and meta-analyses have suggested these potential benefits, particularly in patients presenting with shockable rhythms, indicating associations between early coronary angiography, coronary revascularization, and improved survival post-CA [3,12,13]. However, the recent Coronary Angiography after Cardiac Arrest without ST-Segment Elevation (COACT) trial [14] and Angiography after Out-of-Hospital Cardiac Arrest without ST-Segment Elevation (TOMAHAWK) trial [15], large randomized controlled trials, demonstrated no survival benefit of coronary angiography in patients with ROSC after CA without ST-segment elevation on ECG. The substantial contrast in the outcomes observed compared to previous observational studies has sparked the hypothesis that only certain subgroups of OHCA patients with an increased likelihood of acute coronary occlusions may derive a greater benefit from early coronary angiography. Consequently, further refinements in terms of selecting the appropriate patients for immediate coronary angiography seem necessary.

Previous studies have shown that initial shockable rhythm, cardiac troponin I, and post-resuscitation ECG are poor indicators for identifying patients who would require subsequent intervention. Moreover, considering only traditional risk factors appears insufficient, as our data found: only diabetes had a significant statistical association with significant coronary lesions. This may account for the multivessel calcification seen in diabetic patients, even though arterial hypertension, smoking, and dyslipidemia are also important players in the development of atherosclerotic plaque. In contrast, the quantification of CAC has emerged as a more powerful predictor of adverse cardiovascular events [16,17]. While CAC scoring is traditionally carried out using ECG-gated CT with standard reconstruction and acquisition parameters, calcium deposition within the coronary arteries can also be identified on non-gated chest CT scans [10,18,19]. Despite potential motion artifacts affecting the assessment of coronary calcium on non-gated CT, published data suggest a strong correlation between the CAC scores obtained from non-gated chest CT scans and those from formal CAC assessments [20,21]. A recent study conducted at our center further confirmed this correlation, showing a significant relationship between coronary angiography performed for suspected acute coronary syndrome and CAC documented in previous non-gated chest CT scans [11].

The current study evaluated the correlation between coronary angiography performed due to acute coronary syndrome suspicion in patients with OHCA and CAC documented in a non-gated chest CT. Coronary angiography documented a relatively low prevalence of significant lesions in coronary angiography in this cohort (40%) compared with landmark trials where ~60% patients had significant disease. This could be explained by a possible selection bias, since a non-negligible proportion of patients were directly sent to the catheterization laboratory with no previous or subsequent CT evaluation and were therefore excluded from this analysis. There was an expected association with comorbidities such as diabetes. Our PCI rate (80%) was consistent with previous reports, which have shown rates ranging from 53% to 95% [3,22,23]. The considerable variation in PCI rates across studies may be attributed to differences in the definitions of significant coronary artery disease utilized.

Irrespective of whether quantitative or qualitative assessment methods were employed, patients with significant lesions on coronary angiography exhibited notably higher CAC scores compared to those without significant lesions. Additionally, they displayed significantly elevated CAC scores for the corresponding vessels, except for the left main artery. Delimitating coronary artery calcium in the left main artery poses a challenge due to the imprecise identification of calcification at its bifurcation from the LAD or CX arteries, and beam-hardening artifacts from nearby structures like the aorta or valve annulus can lead to the underestimation of calcium burden in LM coronary. Additionally, due to its small length and surface area, absolute CAC scores are lower than in longer epicardial vessels, accounting for the lack of statistical difference seen between the CAC scores in both groups.

Although CAC provides insight into the presence and burden of coronary atherosclerosis and has an impact on long-term outcome, it does not allow for the identification of obstructive disease [9]. However, the mere presence or absence of CAC provides substantial value in cardiovascular risk stratification, holding important information in terms of coronary artery disease probability. It serves as a straightforward and easily reproducible scoring method requiring minimal additional review time, applicable across various CT protocols without the need for specialized software. In particular, the absence of CAC indicates patients who are at low risk of coronary events. In contrast, the detection of coronary calcium translates into the presence of atherosclerotic coronary artery disease, acting as a biomarker of disease burden and allowing for the better directing of patient care. In fact, finding CAC would permit the avoidance of time-consuming invasive procedures and allow for neuroprotection to be focused on and the causes of CA beyond acute coronary syndrome to be identified and addressed.

The majority of the chest CT reports in the current study did not mention the presence or absence of CAC. This observation aligns with the findings previously documented by Williams et al., who noted that CAC was documented in only 44% of final CT reports among patients with known CAC [24]. In our center, a previous study found that an even lower percentage of CT reports mentioned CAC, with documentation in only 25% of cases [11]. Implementing the routine reporting of CAC on non-gated chest CT scans in patients with OHCA could serve as an indicator of the likelihood of coronary artery disease, thus potentially guiding the decision for early coronary angiography. In addition to raising awareness among radiologists and cardiac imaging specialists regarding the importance of reporting these findings, it is equally crucial to emphasize their significance to clinicians.

## 5. Limitations

This study has several limitations. Firstly, the study population was relatively small and derived from a single institution, necessitating the confirmation of findings in a larger, multicenter population. Secondly, there exists a risk of patient selection bias, as only patients who underwent non-gated chest CT with non-enhanced acquisitions and subsequently underwent coronary angiography were included. Additionally, patients with a history of previous PCI or CABG were excluded from the analysis, limiting the generalizability of the results to this population. Additionally, some patients were evaluated with a 16-slice multidetector CT system, which can limit spatial resolution and influence CAC score estimation. Lastly, the challenge of accurately delimitating CAC is acknowledged. A common pitfall is the inclusion of calcifications from other anatomical structures such as the aortic valve or mitral annulus. Furthermore, delineating calcium in the left main trunk may be challenging due to the imprecise identification of calcification at its bifurcation from the LAD or CX arteries.

## 6. Conclusions

The assessment of CAC in non-gated chest CT scans proved to be feasible and displayed a robust correlation with the presence, severity, and location of coronary artery disease. These results suggest that integrating CAC assessment into the diagnostic pathway for OHCA survivors could aid in identifying those who would benefit from early coronary intervention, tailoring the diagnostic approach—by avoiding time-consuming invasive procedures and identifying and addressing the causes of CA—and thus better directing and personalizing patient care. However, further prospective studies are warranted to validate these findings and refine the role of CAC assessment in guiding clinical decisions for OHCA management.

## Figures and Tables

**Table 1:** Comparison of demographics for patients with and without significant lesions on coronary angiography.

	Total (n = 44)	Patients with No Significant Lesions on CAG (n = 29)	Patients with Significant Lesions on CAG (n = 15)	*p* Value
Age, mean (±SD)	60 (±13)	59 (±14)	64 (±11)	0. 806
Gender—male, n (%)	31 (70)	20 (69)	11 (73)	1
**Comorbidities**				
Arterial hypertension, n (%)	23 (52)	13 (45)	10 (67)	0.213
Dyslipidemia, n (%)	14 (32)	7 (24)	7 (47)	0.177
Diabetes mellitus, n (%)	8 (18)	2 (7)	6 (40)	0.013
Chronic kidney disease, n (%)	5 (11)	2 (7)	3 (20)	0.319
Smoking, n (%)	12 (27)	7 (24)	5 (33)	0.722
Cerebrovascular disease, n (%)	2 (5)	2 (7)	0	0.540
Peripheral artery disease, n (%)	3 (7)	2 (7)	1 (7)	1
Atrial fibrillation, n (%)	7 (16)	4 (14)	3 (20)	0.675
Family history of SCD, n (%)	1 (2)	1 (3)	0	0.620
**CA location**				
Home	19 (43)	13 (45)	6 (40)	0.191
Healthcare institution	3 (7)	3 (10)	0	
Other public spaces	12 (27)	7 (24)	5 (33)	
Unknown	10 (23)	6 (21)	4 (27)	
Witnessed CA	42 (95)	28 (97)	14 (93)	1
No-flow time	4.1 (±7.1)	3.7 (±7.8)	5.1 (±5.7)	0.325
Low-flow time	18.4 (±13.8)	16.8 (±14.7)	21.9 (±11.4)	0.065
CA rhythm				0.913
Shockable rhythm	26 (59)	18 (62)	8 (53)	
Non-shockable rhythm	10 (23)	6 (21)	5 (33)	
Unknown	8 (18)	5 (17)	2 (13)	
GCS immediate after resuscitation, median (Q1–Q3)	4 (3–7)	4 (3–7)	3 (3–5)	

CA, cardiac arrest; CAG, coronary angiography; GCS, Glasgow coma scale; SD, standard deviation; SCD, sudden cardiac death. Chronic kidney disease (CKD) defined as eGFR < 60 mL/m^2^/1.73 cm^2^ or the need for renal replacement therapy.

**Table 2:** Coronary artery calcium scores in the principal coronary vessels—left main trunk, left anterior descending, left circumflex, and right coronary arteries—categorized based on the presence of significant stenotic lesions identified on coronary angiography.

	CAC Score, Mean (+SD)		
	Patients Without Significant Lesions on CAG	Patients with Significant Lesions on CAG	*p* Value
Left main artery	2.9 (+10.1)	26.7 (+29.9)	0.097
Left descending artery	53.2 (+151)	786 (+376.5)	<0.001
Circumflex artery	26.6 (+67.7)	317.4 (+244.8)	<0.001
Right descending artery	24.5 (+109.7)	469.3 (614.9)	0.01

CAC, coronary artery calcium; CAG, coronary angiography.

## Data Availability

All data are available upon request.

## Abbreviations

Area under the curve	AUC
Cardiac arrest	CA
Circumflex artery	CX
Computed tomography	CT
Coronary artery bypass graft	CABG
Coronary artery calcium	CAC
Electrocardiography	ECG
Left anterior descending artery	LAD
Left main artery	LM
Out-of-hospital cardiac arrest	OHCA
Patient Archiving and Communication System	PACS
Percutaneous coronary intervention	PCI
Receiver-operating characteristic	ROC
Return of spontaneous circulation	ROSC
Right coronary artery	RCA
Standard deviation	SD
